# Long Covid: Online patient narratives, public health communication and vaccine hesitancy

**DOI:** 10.1177/20552076211059649

**Published:** 2021-11-29

**Authors:** Esperanza Miyake, Sam Martin

**Affiliations:** 1 Chancellor’s Fellow, Department of Journalism, Media and Communication, 3527 University of Strathclyde, Glasgow, Scotland G4 0LT; 2 Digital Sociologist and Big Data Analytics Research Consultant: Ethox Centre, Nuffield Department of Population Health, Big Data Institute, 6396 University of Oxford, Li Ka Shing Centre for Health Information and Discovery, Oxford OX3 7LF, United Kingdom

**Keywords:** Covid-19, Long Covid, social media < media, health communications < general, online < general, internet < general, self monitoring < personalised medicine

## Abstract

**Introduction:**

This study combines quantitative and qualitative analyses of social media data collected through three key stages of the pandemic, to highlight the following:

- ‘First wave’ (March to May, 2020): negative consequences arising from a disconnect between official health communications, and unofficial Long Covid sufferers’ narratives online.
- ‘Second wave’ (October 2020 to January 2021): closing the ‘gap’ between official health communications and unofficial patient narratives, leading to a better integration between patient voice, research and services.
- ‘Vaccination phase’ (January 2021, early stages of the vaccination programme in the UK): continuing and new emerging concerns.

**Methods:**

We adopted a mixed methods approach involving quantitative and qualitative analyses of 1.38 million posts mentioning long-term symptoms of Covid-19, gathered across social media and news platforms between 1 January 2020 and 1 January 2021, on Twitter, Facebook, Blogs, and Forums. Our inductive thematic analysis was informed by our discourse analysis of words, and sentiment analysis of hashtags and emojis.

**Results:**

Results indicate that the negative impacts arise mostly from conflicting definitions of Covid-19 and fears around the Covid-19 vaccine for Long Covid sufferers. Key areas of concern are: time/duration; symptoms/testing; emotional impact; lack of support and resources.

**Conclusions:**

Whilst Covid-19 is a global issue, specific sociocultural, political and economic contexts mean patients experience Long Covid at a localised level, needing appropriate localised responses. This can only happen if we build a knowledge base that begins with the patient, ultimately informing treatment and rehabilitation strategies for Long Covid.

## Introduction

The term ‘Long Covid’ (and its variations, ‘long term Covid-19’, ‘long-haul Covid’ or ‘post-Covid-19’) gained more widespread official recognition in the latter half of 2020.^1^ This coincides with the time when key international bodies – such as the World Health Organization (WHO) on 21 August 2020,^2^ and the Centre for Disease Control and Prevention (CDC) on 13 November 2020^3^ – finally acknowledged the condition ‘officially’ through reports, webpages and services dedicated to providing more information, support and/or practical advice on Long Covid. Most recently in February 2021, Anthony Fauci essentially renamed and medicalised Long Covid as ‘postacute sequelae of Covid’ (PASC).^4^ Whilst such officialisation processes might seem like the natural result of more people reporting Long Covid after significant time passing since the initial outbreak in the beginning of 2020, on the contrary, patients and researchers had been fighting to get the term and condition acknowledged as far back as early Spring 2020.^5–7^ In this paper, we use the term ‘Long Covid’ to include all discussions of similar terminology surrounding Long Covid, not only Long Covid itself.

Indeed, pre-dating WHO and CDC's public health communications, there were journalists (often sufferers of Long Covid – sometimes known as ‘Long Haulers’ – themselves),^8^ online social media patient narratives, grass-roots patient-led activist initiatives,^9–11^ and a relatively small group of researchers/academics: all actively highlighting the need for more research, information, medical acknowledgement (not to mention a stop to what online discourse referred to as ‘gaslighting’ from uninformed GPs), and greater clarity surrounding treatment and rehabilitation in official health communications. Although Long Covid has now been increasingly recognised and ‘officialised’ since the end of 2020, we now face new concerns regarding vaccines and new SARS-CoV-2 strains. Moving forwards, it is crucial we learn from how initial discrepancies between ‘unofficial’ and ‘official’ accounts of Long Covid led to some negative impacts for the Long Covid community.

In the words of WHO, ‘much is still unknown about how Covid-19 affects people over time’.^12^ The Long Covid community's voice had a direct impact on increasing collaboration between patients, researchers and healthcare professionals (HCPs), leading to greater awareness, knowledge and improved health support services for long-term Covid-19 patients.^13,14^ As we continue through further unknowns, it becomes increasingly crucial for every country to begin research at ground level, through listening to patient narratives and understanding these narratives as not just ‘patient accounts’, but as co-producers of scientific knowledge: indeed, as Nature urges, ‘they must always give proper consideration to the voices of people with Covid-19 and their representatives, who have done so much to put Long Covid on the health-research and policy agenda’.^15^

Whilst governmental responses worldwide have had varying levels of success in terms of containment, number of deaths and ‘recoveries’ from Covid-19, what remains the same in all countries is the existence of Long Covid and the increasing number of Long Haulers worldwide. It is imperative to understand that whilst Covid-19 is a global issue, specific socio-cultural, political and economic contexts mean patients experience this global crisis at a localised level that needs an appropriate localised response. This can only happen if we begin by building a knowledge base that starts with the patient. Only then can we begin to understand the missing gaps in knowledge surrounding Covid-19, and what treatment and rehabilitation strategies might look like for those living with Long Covid.

Taking the UK as a case study, in the following, we explore three key areas through quantitative and qualitative analyses of data collected from Twitter, Facebook, Blogs, Forums and other smaller platforms between 1 January 2020 and 1 January 2021. Firstly, by focussing on data collected during the ‘first wave’ of the pandemic in the United Kingdom (March to May 2020),^16^ we highlight the negative consequences that arise when there is a disconnect between official health communications and research, and that of the actual lived realities of those suffering from Long Covid. Secondly, by focussing on data collected during the start of the ‘second wave’ of the pandemic in the United Kingdom (October 2020 to January 2021) we also demonstrate how in closing the ‘gap’ between these previously conflicting discourses (official health communications – unofficial new media and patient narratives), led to a more integrated approach to support, research and services that considered patient experience. Thirdly, by focussing on data collected during the initial stages of the Covid-19 vaccine rolled-out in the United Kingdom (January 2021), we highlight some of the emerging concerns; these will form the basis of our recommendations of areas that need further consideration in research, policy and healthcare practices.

## Methods

We adopted a mixed methods approach involving quantitative and qualitative analyses of 1.38 million posts mentioning long-term symptoms of Covid-19, gathered across social media and news platforms between 1 January 2020 and 1 January 2021 (Twitter, Facebook, Blogs, and Forums). We conducted: discourse analysis of words, and sentiment analysis of hashtags and emojis (please see below for further details). Taking an inductive approach, we triangulated results from our analyses of different data sets in order to conduct an overall qualitative thematic analysis.^17–19^ Once particular key themes were identified, relevant posts were selected for further textual analysis in order to draw out specific issues which informed our overall discussion.^20,21^

### Data collection

During the first and second waves, data was collected across all platforms globally. In the first wave, the majority of data was found on: Twitter (143,000); Facebook (1081); Blogs (220); News posts on social media (174); Reddit (76); Forums (56) and other platforms (20) giving less results. In the second wave, the majority of data was found on: Twitter (510,000 tweets); Facebook (10,300 posts); Blogs (23.900); News posts (29,500); Reddit (17,400); and Forums (10,363 posts). In addition, there were 212,500 mentions of the Covid-19 vaccine within the context of the first UK Vaccine rollout/phase and the effects on Long Covid, with most data found on: Twitter (1640); Forums (325); and Blogs (147). Facebook posts sampled were at the overview level, with the number of posts recorded, but for reasons relating to ethics and restricted data access, full data from closed Facebook groups and pages was not analysed. In this case, open Twitter, Forum and Blog data was used.

All social media data posts were collected using media monitoring software Meltwater.^22^ Meltwater gave us access to all historical and real-time tweets, and a sample of other social media posts. This enabled us to use advanced search terms to search all posts shared within the selected timeline (January 2020 to January 2021). A Boolean search term (see Appendix 1 for terms) – including keywords and hashtags used to refer to people's experiences of long-term symptoms – was adopted to collect posts about the Covid-19 pandemic. We concentrated on Long Covid discussions in the UK from between 1 January 2020 and 1 January 2021. Only posts/discussions focussing on Long Covid were used. The search terms used were created to capture both mentions of Long Covid, and co-related phrases, words, hashtags and sayings related to the experience of Long Covid. However, posts not containing the extended Boolean search terms were not picked up, which means only the sample of posts referencing Long Covid and co-related terms within the Boolean were sampled and processed for discourse, hashtag and emoji analysis. In this respect, a future study encapsulating all conversation threads around Long Covid, including those that do not mention Long Covid directly will add further knowledge to the existing literature.

We filtered for bots and trolls by excluding posts from accounts with less than three followers, and also, where accounts were less than a few days old. However, we acknowledge that it is extremely hard to completely exclude posts by trolls, and furthermore, some posts by trolls may have affected the responses of non-trolls on the topic of Long Covid. Here, future work should refer to Broniatowski et al.^23^ in spotting and tracking the prevalence and impact of trolls within the context of Long Covid discourse on social media.

### Data analysis

#### Discourse analysis – words

Discourse analysis of social media posts (the worded text of tweets, Blogs, and Forums)^24^ was conducted using text network analysis software Infranodus^25^ to measure themes and patterns occurring in discussions around Long Covid. The betweenness centrality of subtopics was also analysed through examination of connections between subtopics that might link different types of conversation clusters together (e.g. discussions of persisting fever that also mention headaches, where mentions of headaches also occur in conversations about low blood pressure).

#### Sentiment analysis – hashtag and emoji

Sentiment analysis is used to measure the positive, negative, or neutral feelings of users of social media platforms towards communication around a certain topic,^26^ which in our study concerns the experience of Long Covid during the Covid-19 pandemic. Emoji and hashtag analysis was conducted through the interpretation of co-occurrence, frequency and discourse analysis (with the software Talkwalker) which involved examining hashtags and emojis within the context of their posts.^27–29^ The IBM (International Business Machines Corporation) Watson™ emotional lexicon was used to measure different types of emotion across both the first and the second waves. Here, we were able to classify where words were used that portrayed sadness, fear, joy, disgust, or anger towards the topic of Long Covid.^30,31^ Whilst it is difficult to accurately measure participants’ true reactions and complex emotions using big data at a more detailed level, our analysis does indicate some preliminary findings that help to establish the basic emotional field of Long Covid sufferers online. Future research could be built upon these findings, using a combination of psychological and textual discourse methods.^27^

Furthermore, we note that in the process of analysing the use of emojis on social media – especially in relation to complex health issues – the differences between individual psychological characteristics, demographics, platforms, cultural backgrounds, and contexts may lead to different and complex understandings.^32,33^ It is well acknowledged in the literature that there are differences in the way that genders use and interpret emojis.^34,35^ For example, although men and women have been found to understand the function of emoji similarly, women were found to use emojis more frequently and positively, while men use less, but a wider variety of different types of emoji, depending on the public or private type of communication.^36,37^ It has also been found that emojis are used with a high degree of context sensitivity, which means that they are exceedingly dependent on their linguistic and textual environment,^38^ whilst more specific and focused emoji have been found to be used in groups where participants are more sympathetic to a particular topic.^39^

Whilst we were able to look at the differences between gender in the form of textual posts, the limitations of our software meant that we were not able to examine the gendered use of emojis in the ways outlined above. With these limitations in mind, our general thematic approach is thus based on analysing emojis within their linguistic and textual environment in the context of Long Covid – which includes demographic data on gender – but not necessarily the gendered practice behind the use of emojis.

### Patient and public involvement statement

There was no patient and public involvement for this study.

## Results overview

**Figure 1.**
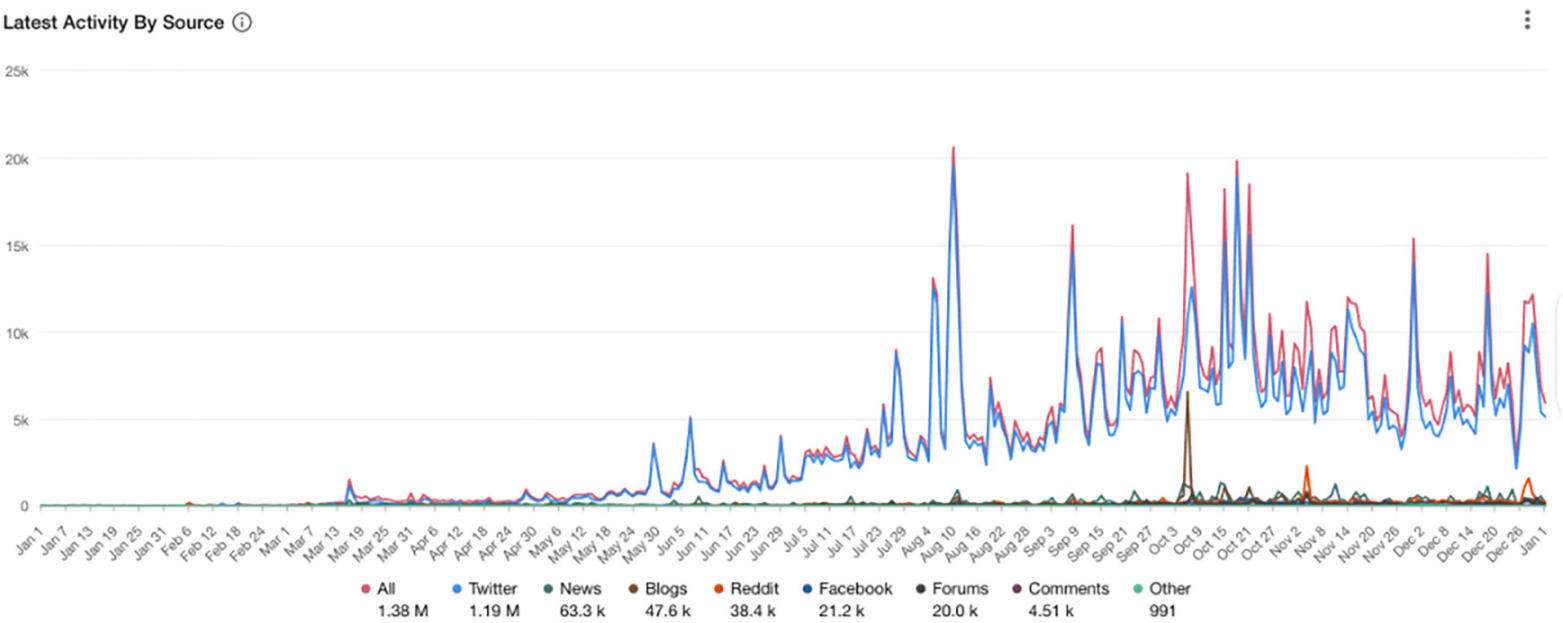
Social and mass media mentions of long Covid and related terms referring to long-term symptoms of Covid-19 (1 January 2020 to 1 January 2021).

**Figure 2.**
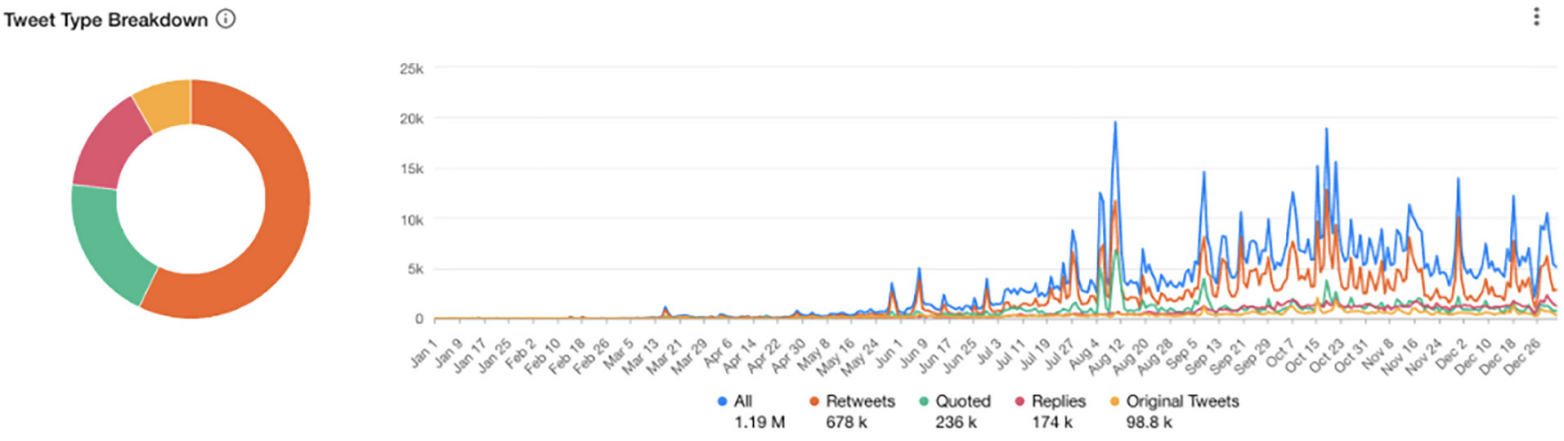
Twitter type of mentions of long Covid and related terms referring to long-term symptoms of Covid-19 (1 January 2020 to 1 January 2021).

Overall in the UK sample, there were a total of 1.38 million posts across social media and news platforms globally (Figure 1). There were 1.19 m tweets, 63.3k news reports, 47.6k Blog posts, 38.4k Reddit posts, 21.2k Facebook posts, 20k Forum posts, 4.51k comments on articles about Long Covid and related terms referring to long-term symptoms of Covid-19, and 991 other types of posts. The majority of Twitter mentions of Long Covid and related terms referring to long-term symptoms of Covid-19 were retweets (678k) (Figure 2), followed by quoted (commented upon) tweets (236k) and 174k replies. Those retweets, quotes and replies were focused on engagement with 98.8k tweets from original/single Twitter users during this period (1 January 2020 to 22 December 2020). Overall, based on followers of each of the users engaging with tweets – the potential reach/impressions of these conversations (possible number of people who may have read these messages) was 177 million.

While there is a complex make up of people, cultures, languages, and geographies posting across social media, the restrictions of the reporting mechanisms of these platforms mean that it can be problematic to retrieve full and accurate information about the complete demographics of all social media posters.^40^ Whilst it was not possible to receive additional demographic data (outside of country location) from news reports, Blogs, Reddit, Facebook, Forums, and other types of posts, it was possible to retrieve data from users who opted to share their gender via Twitter. Additional information about shared gender made up a smaller part of the dataset, with only 285k tweets (42%) clearly classifying gender. This is indicative of the complexity of demographically and geographically tagged data,^41–43^ which we cover further in the Discussion and Conclusion.

Focusing on the UK as a sample of this global data, the demographics of our sample reflects the makeup of people who have actively shared their location and gender via Twitter in the UK. Of those who shared their gender, the majority of people in the UK tweeting about Long Covid during the first wave were: female (59.4%); male (40.6%); with the highest age range of posters being: 25–34 years old (47.4%); 18–24 (29.1%); and 35–44 (17.2%). During the second wave: female (53.4%); male (46.6%), with the highest age range being 25–34 years old (23.9%); 18–24 (46.7%); 35–44 (22%); and 55+ (7.3%). Finally, during the first stages of the Covid-19 vaccination phase: female (24.2%); male (34.3%); data regarding the age-range was not shared and thus unavailable within this smaller group.

## Discussion

### From ‘an internet thing’ to a medical condition: The changing face of Long Covid

Academic literature attempting to specifically define Long Covid began to emerge towards the summer months of 2020.^44–47^ This body of work was crucial in placing Long Covid as a term in its own right – especially as a medical condition – yet there are fewer studies on how Long Covid is understood and experienced by the general population i.e. the Long Haulers themselves.^48,49^ Despite this growing academic body of work (the academic literature sampled for this study spans from its initial emergence mid-2020 until January 2021) – including a published report by the NIHR – the term, ‘Long Covid’ did not always appear in ‘official’ health communications.^50^ The online data we collected relating to the first wave of Covid-19 (1 January to 28 August, 2020) cover the moments when terms like ‘Long Covid’ or ‘Long Haulers’ began to gain significance.

For example, the hashtag #LongCovid appeared on social media on 20 May 2020,^51^ and the term ‘Long Covid’ started to trend on social media in the UK from 25 June 2020 onwards. Similarly, the hashtag #apresj20 (after 20 days) – in reference to people experiencing Covid-19 symptoms for longer than the initial official period of 20 days – was first discussed on social media in early April, mostly likely stemming from a video shared on Twitter on 22 April 2020.^52^ Related, the term ‘Long Haul’, first appeared on a social media group created by those with Long Covid (most notably Amy Watson) in the USA in mid-2020;^49^ and the first hashtag #Longhauler started to gain traction on Twitter from 7 June 2020, with the retweeting of a link to an article referencing the creation of closed ‘Long Haul Covid’ support groups on private Facebook pages.^53^

Out of a total of a sample of 295,000 global social media posts collected during the period of the first wave, the long-term/persistent symptoms of Long Covid were mentioned an average of 1230 times a day. Vibrant discussions of long-term symptoms of Covid-19 began as early as February to March 2020, but ongoing mentions really peaked in volume around 19 May 2020, peaking once again on 5 August 2020 (approximately 11,021 mentions), with continued activity until the end of the sampling period (28 August 2020). The reporting of long-term symptoms that had not resolved after initial onset of viral infection picked-up from May 2020 onwards, when people realised that they were not feeling better within the supposed ‘recovery time’. The official ‘recovery time’ changed throughout the pandemic of course; the fundamental point here is that Long Covid sufferers are diagnosed (if at all) and equally define their own ‘abnormal’ recovery against official time spans at a given moment (more on this later).^54,55^

The high numbers of social media posts alone at the time suggest that ‘how long’ was measured and defined by Long Haulers as occurring much earlier than those officially recognised/quantified by health authorities from June onwards. Before the start of June 2020, Long Haulers were in effect, officially invisible: typical tweets reported Long Haulers either being misdiagnosed/’gaslighted’ by their GPs, those affected by long-term Covid-19 during this period received very little support, information and treatment. Furthermore, during this same period, there was a strong discrepancy between what general ‘official’ definitions of Covid-19 (those appearing in governmental and public health communications),^56^ and ‘unofficial’ definitions as those articulated online by Long Haulers.

Our qualitative analysis of twitter posts collected during the first wave of Covid-19 indicated that the ‘unofficial’, typical Long Hauler narratives at the time followed a consistent pattern that nearly always included the following information: (a) reference to the length of Covid-time endured in days/weeks/months; (b) an articulation of symptoms and reference to an official testing and/or diagnosis (or lack thereof); (c) emotional/intellectual response to Long Covid; (d) sharing of information and resources. These arose mostly from a disconnection experienced by Long Haulers, between the ‘official’ definitions and accounts of these issues, and those of their own. Such a disconnect had clear negative consequences for both individuals and society as a whole, which we explore in the next section. One key example is the use of the hashtag ‘#MedTwitter’,^57^ which during the first wave, was used by Long Haulers to tweet ‘at’ doctors and the rest of the medical establishment, asking for them to take notice of symptoms and not make the same mistake of not listening to patients, that had been made in during and after the H1N1 pandemic.

It was not until the start of the second wave of Covid-19 (October 2020) that the gap between official and non-official narratives began to close, reflecting the start of a period when Long Covid began to be finally addressed or at least acknowledged by official government and health bodies. Between the end of October and the beginning of December 2020, some 1000 tweets used the hashtag #MedTwitter, not just as a mechanism to tweet ‘at’ the medical establishment, but also, as part of an ongoing conversation between groups of Long Haulers, doctors, nurses and academics (themselves affected with Long Covid): all joining the call to investigate the multiple symptoms, experience and response to Long Covid further.

During this period, one particular tweet and link was engaged with (liked and retweeted) over 690 times. This was an academic article shared by a medical doctor, who had contracted Covid-19 and then Long Covid themselves.^58^ This doctor had been engaged in the Long Hauler social media community, and wanted to officially share the importance of medical practitioners listening to patients even in the absence of conclusive testing, as well as the need for patients to feel validated. This is a prime example of both lay and professional (medical/academic) coming together to provide more robust and ‘official’ documentation of Long Covid in more established academic journals and other mediums.

Taking the UK as a case study, in the following we discuss the negative consequences of excluding the patient voice and experience within any ‘official’ research and communications surrounding a new and little-known virus like Covid-19 (as happened during the first wave of Covid-19). We do this by exploring four key areas of initial disconnection between official and non-official Long Covid narratives as identified via patient narratives at the time: a) Covid-19 temporality; b) range of symptoms; c) emotional/intellectual response to Long Covid; d) lack of information and resources. Through data collected during the second wave, we also want to demonstrate how, in the UK, public patient discourses surrounding Long Covid had a direct impact into these four areas and ultimately led to the increasing collaboration between patients, researchers and healthcare professionals (HCPs), which in turn, led to greater awareness and improved health support services for long-term Covid-19 patients.

#### A) covid-19 temporality: How long is long covid?

During the peak of first wave of Covid-19, the official UK health guidelines stated that if one experienced any of the official symptoms (at this point on 12 March ‘a new persistent cough’ and ‘a high fever’),^59^ one must self-isolate for 7 days. If the high fever persisted, one must keep isolating until the fever broke; however, one could emerge from self-isolation if the cough continued. From 30 July, NHS's official public health communication guidelines on self-isolation changed, whereby people were instructed to keep isolating for 10 days and can emerge from self-isolation if one ‘feels ok’ but must continue if one ‘feels unwell’.^60,61^ Such official guidelines defined how long Covid-19 should last, how long one should self-isolate, and how long recovery lasts. Such guidelines provide an official time-frame for everyone to (self)measure the duration of and recovery (and more recently, immunity) period from Covid-19.

However, whilst governmental and health advice is crucial when monitoring, managing and controlling Covid-19, official narratives also produce normative temporalities against which sufferers of Covid-19 are measured and defined. Those displaying symptoms beyond official temporal parameters at best become defined as Long Haulers, or at worst, become diagnosed with another non-Covid-19 condition – sometimes as part of a Covid-19 diagnosis, but more often than not, as an independent diagnosis (e.g. hyperthyroidism, mitral valve disease and pericarditis) – falling within a given ‘appropriate’ time-frame. As such, these dominant temporalities officiate, regulate and define Covid-19 bodies in ways that cause confusion, distress and frustration amongst suffers of both Short and Long Covid: for example, a summary of one Long Hauler's post states, ‘thought I’d be sick for a couple of weeks, it's been 6 months’. Such typical posts indicate there is clearly a challenging emotional (not to mention physical) cost that arises due to the disconnect between the official (a couple of weeks’) and non-official (‘instead it's been X months’) narratives Covid-19.

Similarly, official time-frames given on how fast and/or how long it should take to recover from Covid-19 also have an impact on both sufferers’ wellbeing and the safety of the wider community. For example, there are many online reports of fevers lasting beyond the official expected time: how long should such individuals self-isolate? As such, sufferers’ ability and timing of when they can leave home, when to return to work, when to seek further medical advice will be based on official ‘normal’ Covid-temporalities, which could be detrimental. This brings to the forefront critical questions relating to safety and risk posed on both the individual, as well as society as a whole.

Faced with a mismatch between official recovery times and those experienced in reality, first wave Covid-19 saw the rise of alternative ways in which Long Covid sufferers self-measured, self-monitored, self-documented and self-expressed their condition through counter-temporal narratives. In the absence of a clear temporal definition of Long Covid (when does Covid-19 become Long Covid?),^62^ typical online narratives contain attempts to both quantify and qualify the Covid-19 Self.^63–65^ Most online posts at the time begun with either the number of days an individual had suffered from Covid-19 (e.g. ‘Day 135’) – a continuous temporal articulation, with no ‘cut-off’ to Covid-19 – or reference to the exact date/month when their symptoms began, often in the form of selfies with people holding placards.^66^

As summer 2020 passed by, more and more (self)documented stories emerged not just in social media but the wider popular media discourse that have helped to qualify the Covid-19 self as a body living through a continuous and unbroken pandemic temporality: in the UK, *The Guardian* ran a number articles on Long Covid and Long Haulers,^67–70^ as did the BBC.^71–73^ Such discourses provided a means to narrativise alternative autobiographical temporal trajectories;^74^ these news and social media discourses were crucial in raising awareness of Covid-19 as a cumulative, rather than finite temporal condition.

Finally, when vaccines began to be rolled out throughout various countries in 2021, in the UK, there were new concerns emerging regarding another kind of Long Covid temporality: the timing of vaccines. Discussion surrounding vaccines began to emerge between October and December 2020. Social media discourse indicates that the question of ‘vaccine hesitancy’ stemmed from concerns regarding: vaccine safety for those experiencing Long Covid symptoms, especially concerning the body's immune response to mRNA and other vaccine adjuvants/micro-constituents; and vaccine safety for those who suffered Covid-19 badly first time round, with fears about negative immune response to Covid-related microconstituents in vaccines (Covid-19 Symptom Study).^75^ Links were shared to NHS websites addressing the timing based from these concerns: ‘Should I get vaccinated if I have already had Covid or I am suffering from ‘Long Covid’?’^76^ The advice to this question was to consult with GP: ‘Where you are suffering significant ongoing complications from Covid-19 you should discuss whether or not to have a vaccine now with a clinician.’ (Covid-19 Symptom Study (2020)) Again, the lack of clarity around time and timing was a crucial factor in Long Covid suffers’ wellbeing.

#### B) individual articulation of symptoms: making long covid a collective phenomenon

Another area where there was a marked disconnect between official Covid-19 narratives and those experienced by Long Haulers was in the articulation of symptoms. On the one hand, ‘officially’ recognised symptoms were used to (self) diagnose Covid-19: at the peak of the pandemic, these were communicated in the UK as a ‘new and continuous cough’ and ‘high fever’; as from 18 May 2020,^77^ ‘loss or change to your sense of smell or taste’ was also included in the criteria, as well as other secondary symptoms (Flu-like with no fever; Flu-like with fever; Gastrointestinal; Fatigue (severe level one); Confusion (severe level two); Abdominal and respiratory (severe level three).^78^ However, throughout the first wave, Long Covid sufferers reported a whole other range of symptoms which did not fall under the official Covid-19 criteria,^79^ something that has also been specifically observed and highlighted in the *Covid-19 ‘Long Hauler’ Symptoms Survey Report*.^80^

Online narratives of Long Covid showed a much wider gamut of symptoms. This is a point that has been increasingly raised by doctors and researchers alike. Furthermore, social media posts showed examples of the negative consequences to ignoring the gap between official and non-official symptom-based definitions of Covid-19: some had been misdiagnosed; some had had existing chronic conditions collapsed into Covid-19; others had been dismissed by the medical profession altogether. The one commonality is that most patients seemed to be treated/diagnosed on an individual basis whenever their symptoms did not ‘match’ official Covid-19 guidelines.

Whether contagious or not, an epidemic has a sort of historical individuality, hence the need to employ a complex method of observation when dealing with it. Being a collective phenomenon, it requires a multiple gaze; a unique process, it must be described in terms of its special, accidental, unexpected qualities.^81^

If the ‘medical gaze’ is directed through a very narrow symptomatic lens – just three criteria for Covid-19 – then all other symptoms, knowledge and diagnoses become individualised, rather than being considered as a collective phenomenon of the singular epidemic. Individual Long Hauler narratives on social media brought into public existence the very concept of Long Covid. But such a discursive production of Long Covid should not be viewed as just an ontological matter; individualised articulations of symptoms are part of a *collective* phenomenon that suggest a more multiplicitous gaze needs to be adopted to understand the sheer range of Covid-19. As Stewart describes in his article written as a Long Hauler, ‘the isolation of *experience* is like suffering the whole thing a second time’.^82^ As such, understanding how and why Long Covid needs to be a collective, rather than individualised issue becomes crucial.

**Figure 3.**
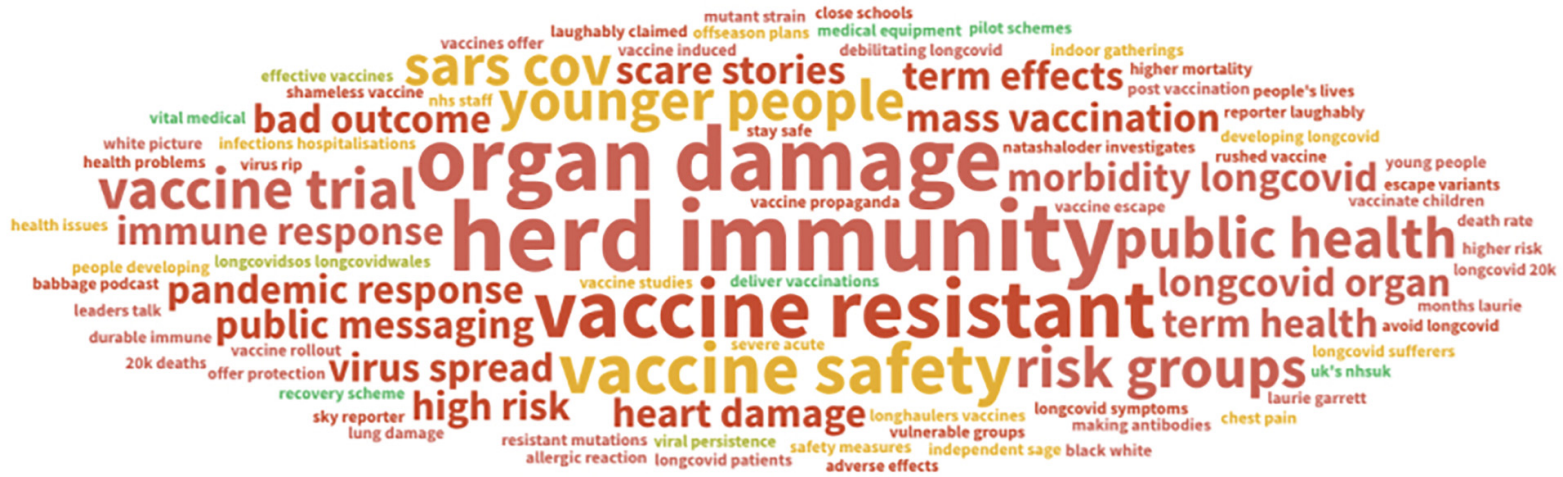
Key topics of concern regarding herd immunity and growing numbers of long haulers, symptoms of long Covid and related terms referring to long-term symptoms of Covid-19, and the impact of the Covid-19 vaccine(s).

The question of the individual versus collective became even more of a key concern when vaccines were being rolled out at the start of 2021. As can be seen from Figure 3, social media discourse analysis indicates that the idea of ‘herd immunity’ is tied to questions of collective effectivity; related are the concerns around what collectivities are being identified (‘younger people’; ‘at risk groups’). The prioritisation of certain demographic categories has raised concerns around delays in getting vaccines – directly relating to ‘official’ temporalities – and within this supposed ‘herd’, where does the ‘Long Covid’ community fit? The boundaries are slippery. For some, younger adults/younger people seem to be put in at a higher risk of developing Long Covid and long-term organ damage, and hence part of discussions surrounding Long Covid. For others, Long Covid is defined as a ‘risk group’ in itself, with regard to ongoing symptoms potentially being exacerbated with reinfection. If Long Haulers were not classified as an ‘extremely clinically vulnerable’ risk group within the vaccine criteria and were lower down on the vaccine priority list, they would be more vulnerable to reinfection, and not possibly benefit from herd immunity.

#### C) affective long covid: mapping the emotional field

There were challenging emotional consequences of being misdiagnosed and/or dismissed by the medical professions on grounds that an individual does not fit the ‘normal’ criteria for Covid-19. As found in our Twitter sample, there is a real emotional cost of not just suffering from Long Covid, but being a victim of ‘gaslighting’ from GPs. If we are to provide better support and care for Long Covid sufferers, recognising the Long Hauler as an informed patient is crucial. In the following, we map out the emotional field of Long Haulers through an analysis of the most used co-related hashtags in posts about Long Covid experiences, and an analysis of emojis in order to explore how they are used to express emotions towards the symptoms/self-care of Long Covid.

**Figure 4.**
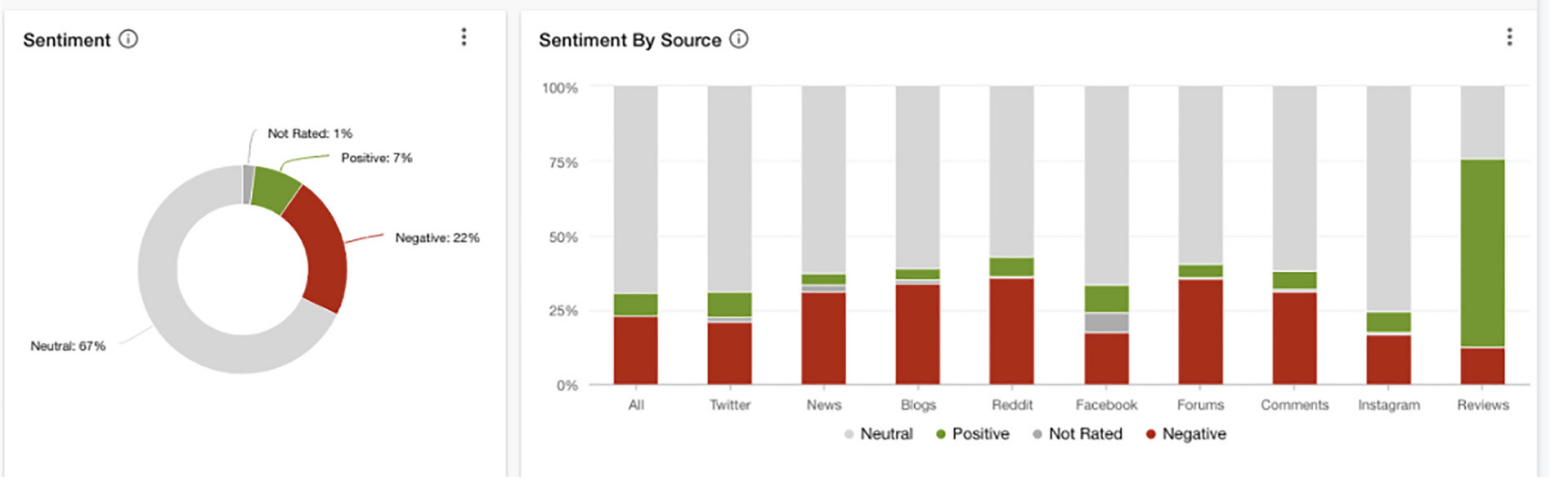
Sentiment by source regarding mentions of Long Covid and related terms referring to long-term symptoms of Covid-19 (1 Jan 2020 to 1 Jan 2021).

Whilst retweeted and reposted news stories (without any additional commentary or human sentiment added) were classed as neutral across networks (Figure 4), the overall sentiment towards the topic of rates of Long Covid was negative at 22%, with just 7% of all posts across social media holding positive keywords. Reddit discussions held the most negative sentiment (35% of Reddit posts), closely followed by reported Blogs with additional commentary (34% negative Blog posts), news posts (33%) Forum posts (33% of all Forum posts), Tweets (23% of all tweets) and Instagram posts (17% of Instagram posts).

**Figure 5.**
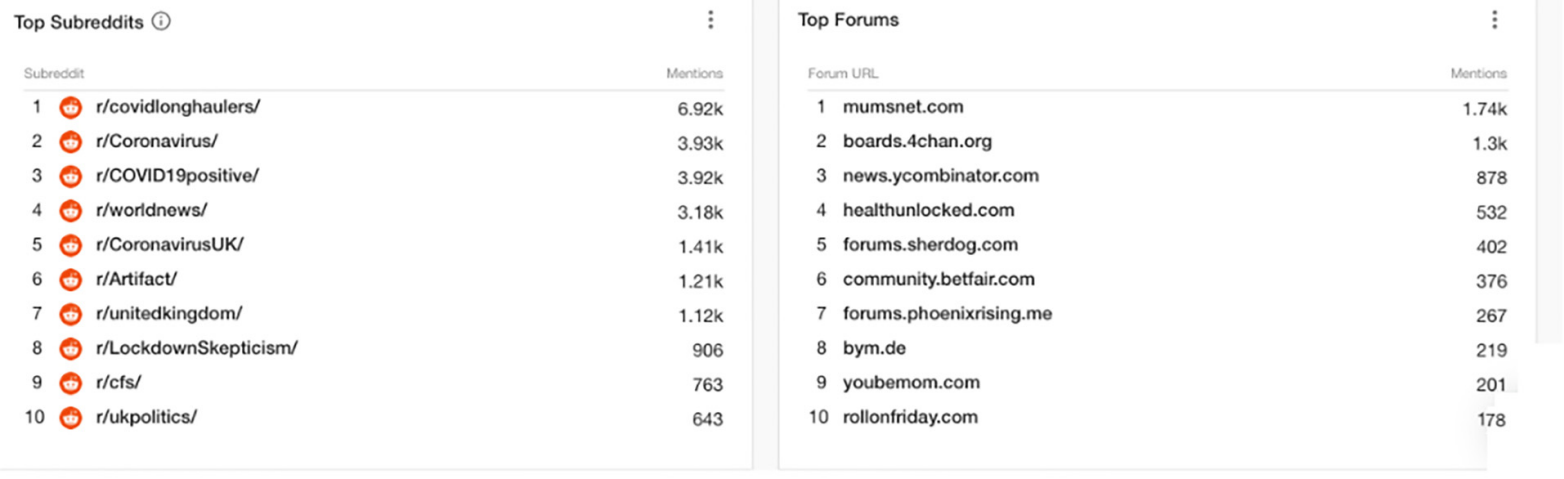
Reddit and Forum mentions of long Covid and related terms referring to long-term symptoms of Covid-19 (1 Jan 2020 to 1 Jan 2021).

The top 10 Reddit groups^83^ that discussed Long Covid and related terms referring to long-term symptoms of Covid-19 included: those directed specifically at Long Haulers; those who had tested as ‘Covid Positive’; those focussed on the symptoms of Chronic Fatigue Syndrome (CFS); and those discussing facets of ‘Lockdown Scepticism’. Top Forums discussing Long Covid included popular Blogs such as Mumsnet,^84^ HealthUnlocked,^85^ YouBeMom,^86^ 4Chan,^87^ Sherdog,^88^ and Betfair^89^ (Figure 5).

**Figure 6.**
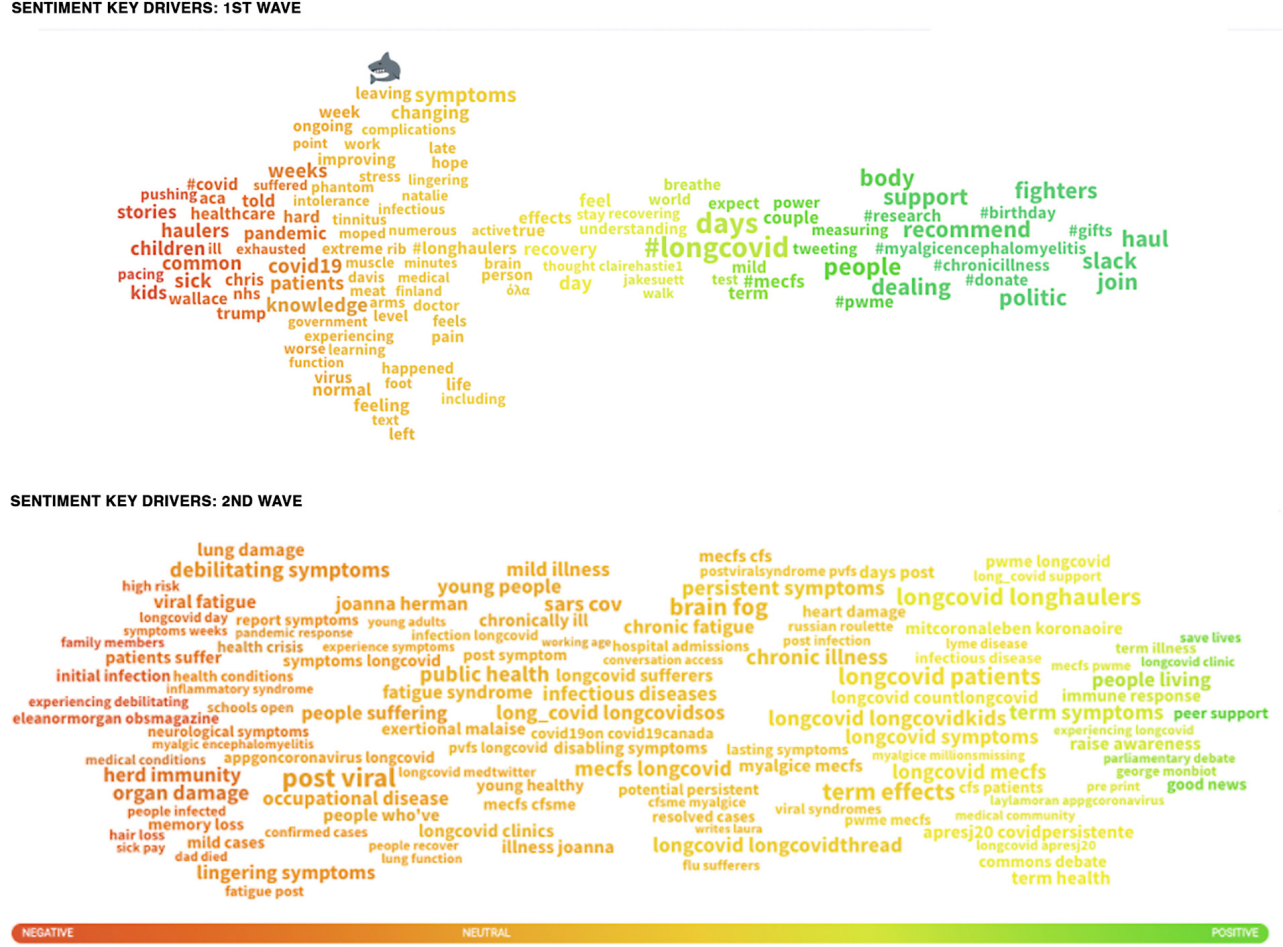
Sentiment regarding mentions of long Covid and related terms referring to long-term symptoms of Covid-19, first and second wave (1 Jan 2020 to 1 Jan 2021).

During the first wave, the majority of sentiment around Long Covid was neutral, alongside the increase of news articles carrying more neutral headlines (e.g. ‘*New national study on long-term health impacts of Covid-19 launched*’, or ‘*Survey reveals impact of long-term Covid-19 symptoms on patients and doctors*’). Here, the emerging occurrence of Long Covid was shared between 29 July and 16 August During this period, the rest of social media sentiment mentions – including posts by those experiencing Long Covid and news headlines – was overall negative (*n* = 27% posts) between the end of May and the end of August 2020. In comparison, 11% were positive posts, many of which discussed the growth of Long Covid support groups and knowledge about self-care. Therefore, analysis of the emotional field of Long Covid experiences on social media shows that whilst there was a peak of neutral sentiment between July and August (Figure 6), the overall emotional sentiment towards Long Covid between June and August 2020 was negative (Appendix 1).

Figure 6 shows a coloured word cloud, which visualises the range and mix of sentiment around specific keywords used with reference to Long Covid and related terms referring to long-term symptoms of Covid-19. Some words are not clearly red for negative, orange for neutral, or green for positive. Instead, they are coloured with a gradient of red, moving through to orange then yellow, and then to green. This shows a mixed range of sentiment towards how Long Covid was discussed on social media, and peoples’ reaction to events as they shared or quoted original posts discussing/describing worry about the lingering and sometimes changing types of symptoms of Long Covid: from muscle pain, overall pain, brain fog, phantom smells, tinnitus, to feeling exhausted and many more; these accompanied by their counting of days/weeks/months of their symptoms.

There were also discussions around the lack of knowledge about recovery from Covid-19. Within the green and positive keywords, people spoke favourably of how helpful joining recommended support communities were, especially those on Slack (see the words ‘body’ and ‘politic’ at the top and bottom of the green wordcloud section). In addition, we found discussions of similarities that Long Covid had to other chronic illnesses, such as Chronic Fatigue Syndrome/ME (Myalgic Encephalomyelitis). Therefore, discourse analysis looking at the sentiment of keywords used with reference to Long Covid and related terms referring to long-term symptoms of Covid-19 shows two thirds of all posts (295,000) included a mixture of negative to neutral (Figure 6: red-orange-yellow), with one third of keywords using positive terms.

Analysis of the second wave (October 2020 to 1 January 2021) (Figure 6) also shows a mixed range of sentiment towards Long Covid and related terms referring to long-term symptoms of Covid-19, and people's reaction to events as they retweeted or quoted original tweets discussing their anxiety over how Long Covid was handled during the first year of the pandemic and various lockdowns. In the second wave, the majority of negative to neutral/ambiguous sentiment (Appendix 1) centred around the seriousness of symptoms and worry about reports of organ damage during different periods of the 2020 pandemic. In particular, there was a focus on: initial infection; the length of symptom experience; the prevalence of chronic fatigue symptoms; comparison with similar chronic illnesses such as ME/CFS; the experience of brain fog; neurological symptoms; worry about the growing number of young adults and kids/children reporting Long Covid symptoms; rising reports of heart damage; and discussions of Long Covid as a post viral syndrome. Neutral to positive sentiment centred around: optimism of newly opened NHS programmes/clinics focussed on supporting Long Haulers; parliamentary debate of Long Covid; increased scientific studies focussed on Long Covid; growing peer support that helped with the quantifying of symptoms; and more wide-ranging qualitative analysis, in turn, being shared with academics and NHS support services.

Of the 42% of people that indicated their gender on their Twitter accounts across both the first and the and second waves, male and female sentiment regarding the experience and symptoms of Long Covid were very similar. Whilst slightly more females expressed negative sentiment (women = 45%, men = 34%), overall, positive sentiment towards Long Covid was low: just 15% positive posts for both men and women, with the breakdown showing more women expressing positive sentiment in this case (women = 8%, men = 6%) sentiment. Neutral sentiment was almost the same (women = 3.7%, men = 3.5%).

Referring to the IBM Watson emotional lexicon, sadness was the overall emotion expressed across both gender groups (66.7%). The majority of emotional words were used by women, who used emotive words 57% of the time, while men used emotive words 42% of the time. The following breakdown of emotional words used by gender is: sadness: male (43%), female (57%); fear: male (41%), female (59%); joy: male (45%), female (55%); disgust: male (46%), female (54%); and anger: male (46%), female (54%).

However, the perspective on emotion and sentiment changes when looking at the context of when these emotive words were used. Perhaps a reflection of more women being diagnosed with Long Covid,^90^ the majority of original posts discussing Long Covid on Twitter were started by women (20%), whilst less men started threads about Long Covid (14%). Women also wrote more quoted replies, with 7% quoting other posts in their replies, compared to 3% of men. Women also made more direct replies in comment threads (30%), although males replied at a higher rate than they had in previous categories (26%).

##### i. Emojis

**Figure 7.**
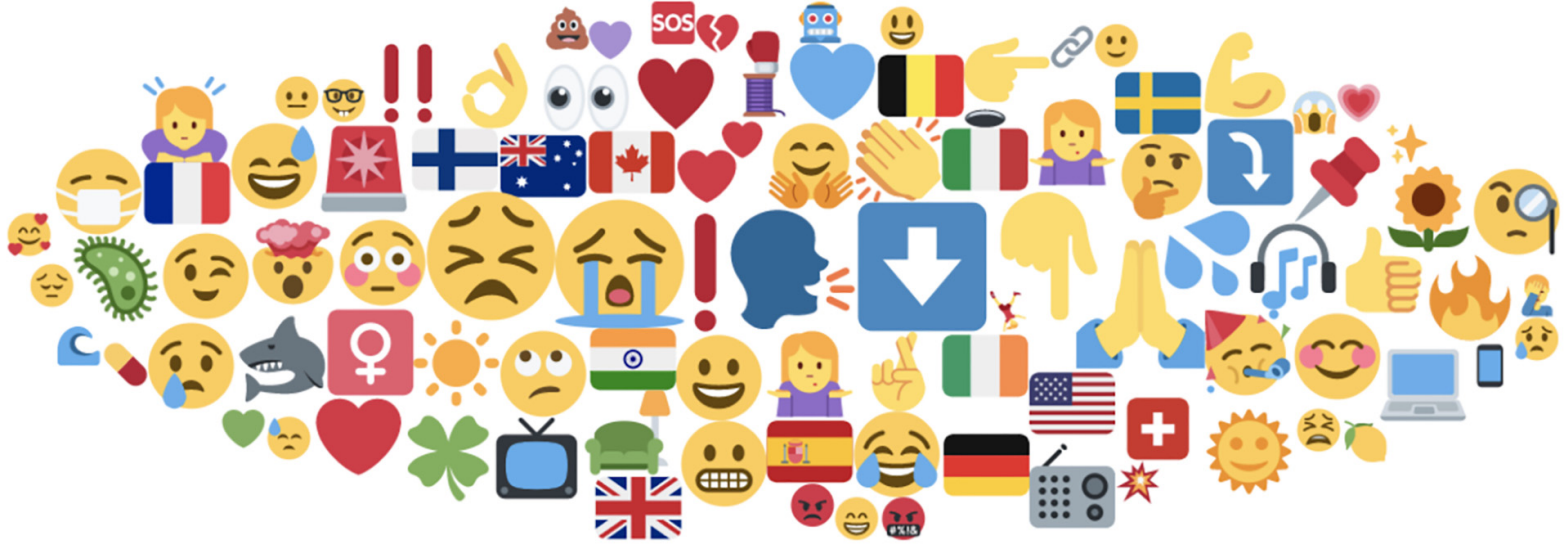
Collection of top emojis used in relation to posts mentioning long Covid and related terms referring to long-term symptoms of Covid-19, and discourse behind them.

From our UK data sample, we found that during the first wave, the majority of emojis used within discussions on Long Covid and related terms referring to long-term symptoms of Covid-19 were symbols that related to the range of emotions of confusion, sadness, crying, alarm, frustration, anxiety, and patients' investigation of the reasons behind Long Covid (as well as celebration at being recognised by GPs or final recovery from Long Covid). These emotions were generally represented by various smiley faces used in posts (Figure 7). Other emojis used included the nationality of Long Haulers, by a range of flags: national identities of Long Covid. Emojis were also linked to symbols for sending support (e.g. shamrock, hearts, sunshine, flowers and praying hands), while others are linked to symbols for alarm, request for support or the hospital (SOS sign, siren and white cross in red square). Finally, some emojis used were symbols/arrows and fingers pointing towards links to articles, videos or other resources shared between users. Therefore, analysis of social media posts finds that emojis were used in quite expressive ways overall.

As to be expected, social media posts thus show that the topic of Long Covid is quite an emotional one, where sentiment, emotive words and expressive all range from negativity towards symptoms suffered, and a determination to share links and knowledge that might help others within the Long Hauler community. In all the actions and performance of the ‘affective patient’ in quantifying, sharing and supporting the experience of Long Covid seems to convey a very much embodied and emotive process.

During the second wave, the majority of emojis used in discussions surrounding Long Covid were symbols showing sadness, crying, sarcastic/disbelieving crying-eyes-laughter and rolling eyes emojis, reports of increased call-outs to emergency services (siren emojis), and the red ‘X’ symbol denoting disagreement with the gaslighting of Long Covid patients (Figure 7). The thinking emoji was used to denote the need to think seriously about taking measures to support those with Long Covid, as well as a sarcastic response to ‘gaslighting’ or not being believed when reporting Long Covid and related long-term symptoms. The praying hands and bicep emojis were mainly used in relation to encouraging those suffering with Long Covid symptoms to stay strong, as well as offers of support to Long Haulers going through particularly trying symptom episodes. The thread/yarn symbol was often used to denote a Twitter ‘thread’ of several tweets, where Long Haulers discussed their experiences or other pressing topics of concern.

Discussions of the virus, masks and vaccines were also represented by corresponding emojis, while country specific experiences or discussion of country specific policies towards Long Covid were represented by country flags: pointing to the regionalised experiences of a global pandemic. Negative to neutral emojis were connected to arrow and pointing finger emojis, often with links to articles, images or other resources shared. The hand-clapping emoji was used both in relation to the clap-for-carers and healthcare workers events happening throughout the pandemic, as well as a sign of congratulations or applause for any progress made by Long Haulers in their experience or management of symptoms, or new research providing new insight into Long Covid. The red circle emoji was used to denote newly reported cases of Long Covid, while the blue heart emoji was used in connection with the #NHSblueheart hashtag as a show of support for NHS workers that were experiencing social media abuse relating to misinformation about increased coronavirus hospital admissions during the second wave.

##### ii. Hashtags

**Figure 8.**
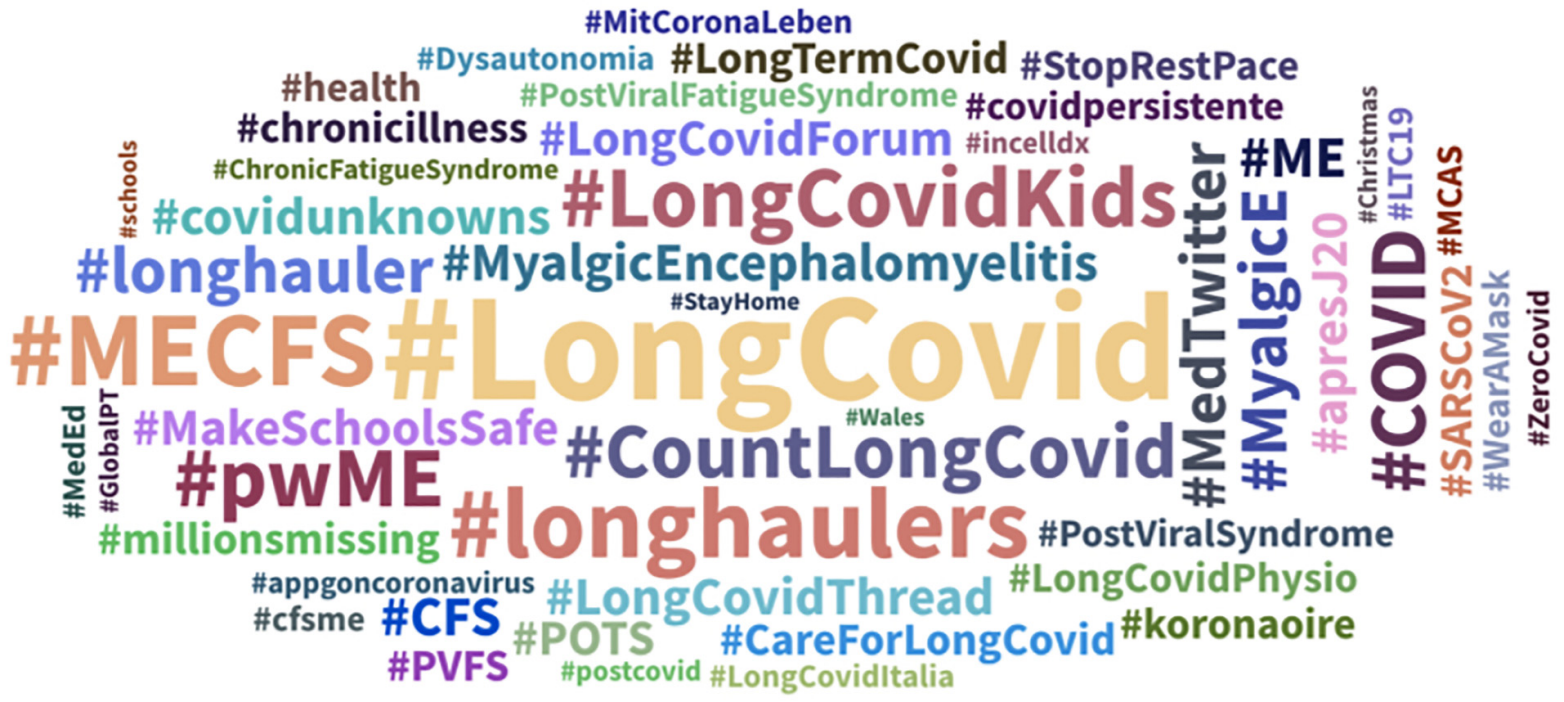
Collection of top hashtags used in relation to posts mentioning long Covid and related terms referring to long-term symptoms of Covid-19 over first and second waves.

Between 1 January 2020 and 1 January 2021 – outside of the main Long Covid hashtags (#LongCovid #CountLongCovid #CovidVaccine and #COVID and #longhaulers) – amongst the top 100 co-related hashtags were ones which focused on the comparison of specific Long Covid symptoms and support groups to long term chronic illnesses, such as Myalgic Encephalomyelitis (M.E) and Chronic Fatigue Syndrome (CFS or MECFS) and Dysautonimia – linked to heart damage and other organ damage (Figure 8). The hashtag #StopRestPace was also used as a way to share advice amongst Long Haulers on how to deal with symptoms that affected their daily lives and ability to cope with work or childcare – most specifically with regards on how to pace themselves, as well as practice different forms of physio (#LongCovidPhysio).

There were also hashtags linked to growing concern about children experiencing Long Covid symptoms (#LongCovidKids), including worry about the effects of post viral fatigue syndrome on children's lives, and the need to make schools safer for children by calling for the wider use of masks within school environments. Other hashtags such as #MedTwitter were used by Long Haulers with specific medical questions/articles that they wanted to ask or share with medically/academically trained Twitter users, who were often also Long Haulers themselves.^91^ Thus, the range of hashtags shared stayed consistent over both the first and second waves, with a focus on the impact of symptoms and links with chronic illnesses. This may be a reflection of there being no major changes for the Long Hauler community outside of investigation within these areas, with a need to understand the progress and treatment of Long Covid alongside research on Covid-19 virus and its continued impact on society.

#### D) sharing information: patient-led research, support and non-medical resources

In the absence of sufficient and widely accessible medical knowledge surrounding Long Covid (as opposed to Covid-19 more generally) – not to mention the competing definitions of Covid-19, as explored earlier – during the first wave of the pandemic, news and social media have taken its role as an important if not the only information resource on Long Covid. Social media has not only become a site for the articulation and documentation of Long Covid from the affective patient, but also where one can find patient-led knowledge through the sharing of resources. This is particularly the case with initiatives like the Body Politic's Covid-19 Support Group,^92^ which birthed a Long Covid-specific Patient-Led Research Collaborative that engages with patient-led research project,^93^ encouraging contributions from all Covid-19 sufferers, as part of a collective strategy of developing a patient-led knowledge base.^94^ We have identified three main types of information Long Haulers share on social media as a direct result of an absence within official channels and domains of knowledge:

- News media: there are a significant number of posts that share news media articles, usually as a way of validating a Long Hauler's experience. This is particularly concerning when news media sources are used in contrast to official diagnoses or guidelines, presented as a more realistic reflection of Long Covid.
- Support network: From the global sample of posts, it was found that quantitative self-tracking information (e.g. number of steps achieved as part of recovery, number of days in pain) often formed a qualitative message of hope to fellow Long Haulers, or a way of sharing experience within a supportive online community.
- Practical hints and tips: the sharing of *practical* advice regarding the use of specific supplements taken to help with recovery (e.g. Glutathione and NAC, Zinc, vitamins C and D), practical tips on how to talk to GPs and other points to help Long Haulers improve their sense of wellbeing.

Towards the end of the first wave of Covid-19, doctors and clinicians (often Long Haulers themselves) began to highlight ‘a frustrating lack of access to appropriate investigations for symptomatic individuals and their doctors due to lockdown and a reduction of services’, and most importantly, how despite the best efforts, ‘many affected individuals have been dismissed with the label of “anxiety” and have endured incredulity and a lack of sympathy or support’.^95^ It was clear that consideration was urgently needed in acknowledging and communicating information regarding Long Covid more effectively. In contrast, during the second wave, there was more recognition of different rates of access to Long Covid clinics and support, as newer scientific studies and NHS support services focused more on the long-term symptoms and specific types of neurological/organ damage suffered by Long Covid patients. As the Covid-19 vaccine(s) began to be rolled out, some of these issues from both the first and second wave continued to be of concern, alongside new ones relating to the lack of clarity and support surrounding vaccines for those living with Long Covid.

## Conclusion

As outlined in the Introduction, there is a promising increase in the number of studies and research on Long Covid and improved Long Covid patient care. However, as our article has hopefully demonstrated, there are detrimental consequences to ignoring patient voices, especially as we move through the process of vaccination and rehabilitation thereafter in a post-pandemic future. Furthermore, as the relatively recent re-naming of Long Covid to PASC demonstrates, there is still clearly a tension between ‘official’ and ‘unofficial’ narratives of Long Covid that may need reconciling.^96^ This is crucial in order to not just understand Long Covid, but Covid-19 itself: from its temporal and symptomatic dimensions, emotional and practical impacts, right through to the way health communication frames the condition and guidelines for Long Haulers and their carers.

Whilst we hope that this article and study opens the way to a more patient-centred approach to Long Covid research, we recognise some limitations which can hopefully inform future research. Firstly, we note that despite Covid-19 being a global epidemic, our research was narrowed down to English-speaking countries that have digital access, infrastructure and literacy to comment on social media. As such, some of the more ideological aspects we discussed – from emotions to questions of identity – are very much socio-culturally specific and as such, are not necessarily global. Similarly, our social media data will not have captured all the various discussions and origins of discussions from different communities – from the medical/scientific community, the post-viral/ME/CFS community, the Long Covid community – around the world, all with different sources of data and understandings of issues relating to health and wellbeing. More research into how constructions and definitions of both Long Covid and Covid-19 differ according to different socio-cultural, geo-political contexts – and those occurring beyond social media – would be appropriate for a global phenomenon.

It should also be noted that whilst our study was partially based on geotagged posts from across social media posts and articles from other media, the limitations of geotagged and demographically tagged tweets and posts should be kept in mind.^50–52^ Drawing on the discussions of the reliability of demographically tagged research,^46^ we note that whilst our data sample is quite large, it retains some bias as users on social media and the small percentage of those that accurately tag their demographics are not representative of the general global or UK population. The consequence is that the demographics indicated in this study do not perfectly mirror the larger population affected by Long Covid.

Secondly, related to the first point, we also recognise that all people – for whatever reason – do not engage with social media more generally. Capturing the voices of those who do not engage with social media and/or online sphere would also be crucial.

Thirdly, to gain an in-depth understanding of Long Haulers’ experiences, emotions and practices would require a further systematic and direct approach i.e. focus groups and/or interviews, which the analysis of short tweets do not necessarily capture in its entirety.

The strengths of our online analysis of Long Covid narratives contributes towards understanding some of the experiential, emotional and practical dimensions of Long Covid, identifying four main areas that need further urgent attention and reconsideration: a) the time-frames assigned to Covid-19; b) the range of symptoms which affects testing/diagnoses; c) the emotional/intellectual impact on Long Haulers; d) lack of resources and information. Whilst services and support groups have begun to emerge, we argue that until official definitions and understandings surrounding Covid-19 are reconsidered, doctors and other services will be limited in their capacity to provide better support, guidelines and social measures for those suffering from Long Covid, as well as their carers. As vaccines are rolled out whilst new strains of Covid-19 spread globally, the next steps we take in dealing with issues relating to patient wellbeing and care – not to mention a greater understanding of Covid-19 – depend on how much we have learnt our lesson in 2020, to begin with the solid foundational knowledge generated by patients with Long Covid, an expansion of more Long Covid support services, and greater collaboration between HCPs and patients.

## Ethical approval

We submitted to a formal ethics review process conducted by the SEC at the University of Strathclyde, which included information relating to relevant criteria outlined by the Consolidated Criteria for Reporting Qualitative Research (COREQ) guidelines. The project collects only publicly available social media data which has been anonymised through various means, notably a Python script to replace all usernames and links with an encrypted tag code. Where discussions involve qualitative analysis of social media posts, these are referred to only in general thematic terms with no attributable direct quotes used anywhere in our article. The SEC determined that our work does not constitute research involving human participants, and thus met the appropriate ethical standards of research.

## Guarantor

EM.

## Contributorship

EM/SM conceived original idea for study. SM designed quantitative data methodology, conducted quantitative data collection and produced data visualisations. EM organised/structured collected data thematically and conducted data analysis. EM wrote: research question/aims and objectives; qualitative discussion, introduction and conclusion. SM wrote: Background; Methodology; quantitative discussion. EM/SM edited together and co-wrote: Abstract. EM sought ethical approval from Strathclyde. EM responsible for admin.

## Appendix 1. Boolean search term for long covid

((‘Long Covid clinic*’ OR ‘Longcovidclinic*’ OR ‘Long Covid Care’ OR ‘Long COVID Physio’ OR ‘LongCOVIDPhysio’ OR ‘Long COVID Recovery’ OR ‘longCovid*’ OR ‘Long Covid’ OR ‘post-COVID-19 syndrome’ OR ‘post-COVID syndrome’ OR ‘post COVID syndrome’ OR ‘LongHauler*’ OR ‘Long Hauler*’ OR covid1in10 OR covid1in20 OR longhaulcovid OR ‘apresj20*’) AND (‘stress*’ OR ‘symptom*’ OR ‘shortness of breath’ OR ‘pain*’ OR ‘tired*’ OR ‘brain fog’ OR ‘fatigue*’ OR ‘fever*’ OR ‘temperature*’ OR ‘insomni*’ OR ‘ache*’ OR ‘#SOB’ OR ‘mental health’ OR ‘pins and needle*’ OR ‘chest pain’ OR ‘chest tight*’ OR ‘concentrat*’ OR ‘can't sleep’ OR ‘insomni*’ OR ‘heart pain*’ OR ‘heart palpitati*’ OR ‘joint pain*’ OR ‘depress*’ OR ‘anxi*’ OR ‘tinnitu*’ OR ‘ear pain*’ OR ‘earache*’ OR ‘sickness’ OR ‘nausea’ OR ‘stomach pain*’ OR ‘stomach ach*’ OR ‘appetite’ OR ‘diarrhoea’ OR ‘headache*’ OR ‘rash*’ OR ‘flare*’ OR ‘high temp*’ OR ‘smell*’ OR ‘taste*’ OR ‘weak*’ OR ‘worr*’ OR ‘vacci*’ OR ‘clinic*’ OR ‘treatment*’))
